# Isolation of Antibiotic-Resistant Bacteria from the Air Plume Downwind of a Swine Confined or Concentrated Animal Feeding Operation

**DOI:** 10.1289/ehp.8910

**Published:** 2006-03-27

**Authors:** Shawn G. Gibbs, Christopher F. Green, Patrick M. Tarwater, Linda C. Mota, Kristina D. Mena, Pasquale V. Scarpino

**Affiliations:** 1 University of Texas Health Science Center, School of Public Health, El Paso, Texas, USA; 2 Department of Civil and Environmental Engineering, University of Cincinnati, Cincinnati, Ohio, USA

**Keywords:** antibiotic resistant, bioaerosols, CAFO, confined/concentrated animal feeding operation, microorganisms

## Abstract

**Objective:**

In this study we evaluated the levels of antibiotic- and multidrug-resistant bacteria in bioaerosols upwind, within, and downwind at locations 25 m, 50 m, 100 m, and 150 m from a swine confined animal feeding operation.

**Design:**

We used Andersen two-stage samplers to collect bacterial samples, the replicate plate method to isolate organisms, and the Kirby-Bauer disk diffusion method to determine antibiotic resistance.

**Results:**

The percentage of organisms resistant to at least two antibiotic classes and all four classes evaluated were, respectively, 2.1 and 3.0 times higher inside (*n* = 69) than upwind (*n* = 59) of the facility. *Staphylococcus aureus* was the most prevalent organism recovered. Concentrations of antibiotic-resistant *S. aureus* decreased with increasing distance from the facility. Using Fisher’s exact methods, the change in distribution of antibiotic resistance profiles for each antibiotic was statistically significant (oxytetracycline, *p* = 0.010; tetracycline, *p* = 0.014; ampicillin, *p* = 0.007; erythromycin, *p* = 0.035); however, this relationship was not seen with lincomycin and penicillin (*p* > 0.05). In addition, the levels of antibiotic-resistant *S. aureus* 25 m downwind were significantly greater than the levels from samples taken upwind from the facility for the same four antibiotics (*p* < 0.05). The percentage of resistant group A streptococci and fecal coliform increased within the facility compared with upwind values for all antibiotics evaluated, except for lincomycin. The percentage of resistant total coliform organisms increased within the facility compared with upwind values for oxytetracycline and tetracycline.

**Conclusions:**

Bacterial concentrations with multiple antibiotic resistances or multidrug resistance were recovered inside and outside to (at least) 150 m downwind of this facility at higher percentages than upwind. Bacterial concentrations with multiple antibiotic resistances were found within and downwind of the facility even after subtherapeutic antibiotics were discontinued. This could pose a potential human health effect for those who work within or live in close proximity to these facilities.

Modern animal husbandry in the United States and other parts of the world has evolved the swine industry from one that was pasture based into a system based predominantly upon confinement and concentration of animals (Perez-Trallero and Zigorraga 1995; Scarpino and Quinn 1998). Most current animal production facilities rely on confined animal feeding operations (CAFOs) and the addition of subtherapeutic doses of broad-spectrum antibiotics to swine feed as a cheaper way to prevent disease and maintain production yields (Witte 1998).

Antimicrobials are known to promote growth in swine and improve the efficiency of feed conversion and can affect bacterial and fungal disease prophylaxis among the confined animals (Davies and Roberts 1999). These treated animals generally gained weight 4–5% faster than other animals not given the antibiotics. Feeding animals antibiotics is associated with the development of antibiotic-resistant bacteria within these animals (Burriel 1997; Nijsten et al. 1996; Threlfall et al. 1993). Antibiotic use within food production animals has raised concern among public health authorities regarding the development of antibiotic-resistant bacteria in dosed animals and the possible subsequent impact on the health of farmworkers and others in proximity to the CAFOs (Witte 1998).

Antibiotics are the leading treatment method for bacterial infectious diseases, which remain the most common cause of death worldwide (McGeer 1998). It is widely accepted that antibiotic-resistant pathogens make clinical treatment more difficult (Takafuji 1977). At local levels, areas surrounding swine production facilities might notice a rise in the difficulty of treating human health problems (Haglind and Rylander 1987). These health concerns include, but are not limited to, respiratory problems, infectious disease, and hypersensitive reactions (DuPont and Steele 1987). Those individuals who live or work in proximity to a facility spreading antibiotic-resistant bacteria could face higher exposures to these organisms (Scarpino and Quinn 1998). Gibbs et al. (2004) recovered antibiotic-resistant organisms known to have adverse human health effects both inside and downwind of the facility. It is an important next step to begin evaluating the distance these organisms can travel within bioaerosols to eventually address public health impact.

The CAFO evaluated in this study was not using subtherapeutic doses of antibiotics at the time air sampling was conducted; however, the animals had received subtherapeutic levels of antibiotics 4 weeks before sampling. The primary objective of this study was to determine the levels of antibiotic-resistant bacteria, including multidrug-resistant bacteria (those resistant to at least two classes of antibiotics) found in air plumes 25 m upwind and 25, 50, 100, and 150 m downwind from a CAFO. These organisms could affect the health of those in proximity to the facility, such as employees. We hypothesized that the quantity of antibiotic-resistant bacteria would show a negative correlation with distance from the CAFO facility, which would support previous research showing that the animals within CAFOs are significant sources of antibiotic-resistant organisms (Gibbs et al. 2004).

## Materials and Methods

### Sample collection

The sampling site, a 4-year-old facility that houses up to 1,000 sows for reproduction purposes, has been described previously (Gibbs et al. 2004; Green et al. 2006). The building is 12 m wide × 60 m long × 3 m high; its sides are concrete to 1 m, with mesh above the concrete to allow air exchange. Computer-controlled shades, located above the mesh, are adjusted depending upon the facility’s internal temperature. The facility employs a chimney ventilation system to draw air through the sides of the building and up through the roof. This system, in conjunction with the shades, cools the hogs and helps maintain the temperature of the building. The facility has a grated floor that allows waste material to fall through into a 1.3-m deep pit that runs the length and width of the facility. Subtherapeutic levels of oxytetracycline were administered to hogs for 2 weeks. The animals were not being given subtherapeutic levels of antibiotics during the study period and had not been exposed to subtherapeutic levels of antibiotics for 4 weeks. The waste material was removed from the pit twice a year and injected into the cropland surrounding all sides of the confinement facility as a source of nutrients; however, at the time of sampling for this study, the injection of waste material had not been done in > 4 months.

The site was sampled four times at different times of the day, with sampling location sampled simultaneously on 16 June 2003 (in the afternoon), 14 July 2003 (in the afternoon), 21 July 2003 (in the morning), and 28 July 2003 (in the evening). The sampling was done at different times of day to accommodate the needs of the facility operator. Methods were adapted from previous studies (Gibbs et al. 2004; Green et al. 2006). All sampling material that could be autoclaved was autoclaved for 15 min at 15 psi and 121°C. Andersen two-stage samplers were sterilized after each use, washed, and then sterilized again before their next use. All other items were disinfected with a 70% ethanol solution after each sampling trip and before the next sampling trip.

We used Andersen two-stage samplers to collect all bacterial samples from the animal confinement facilities. The Andersen two-stage sampler is a cascade impactor that contains 200 orifices for each of the two stages, which separate particles according to their size. The sampler was loaded with plates of tryptic soy agar (TSA; Difco Laboratories, Detroit, MI), an excellent general agar known to have the ability to culture a variety of bacterial microorganisms. The nonrespirable particles approximately 8 μm or larger were deposited on the first petri dish, and the respirable particles of 8 μm down to 0.8 μm were deposited on the second petri dish.

During sampling, the wind direction and wind speed were determined (Davis Vantage Pro weather station; Davis Instruments Corp., Hayward, CA). Air samples were taken immediately upwind of the facility, inside the facility, immediately downwind, and 25, 50, 100, and 150 m downwind. Triplicate samples were taken at each location for quality control. Each sample was taken from the top of a tripod 1.3 m above the ground or floor to simulate the height of the average person. Separate equipment, including a pump (Gast Oil-less Pressure/Vacuum Pump; Gast Manufacturing, Inc., Benton Harbor, MI) and an Andersen two-stage sampler were used for each location on the site. The pump was calibrated to 28.1 L/min before each sampling event. Sampling time varied between 15 sec and 5min, depending on the site’s proximity to the facility, to provide a countable number of colony-forming units (cfu) per plate; samples were taken in triplicate. We followed this procedure for each of the sampling locations. The plates were always handled using aseptic technique to ensure that the air sample was not contaminated and were returned to the laboratory for analysis within 12 hr. In the laboratory, the plates were placed in an inverted position in an incubator at 35°C. The colonies that developed were counted after 24 and 48 hr to determine if the plates were overgrown. After 48 hr of incubation, the plates were inverted and refrigerated at 4°C until they were ready to be used for the replica plate method (Lederberg and Lederberg 1952).

### Isolation and speciation

We used the replica plate method to identify recovered aerosolized bacteria by transferring the bacterial colonies onto a selective medium (Lederberg and Lederberg 1952). The replica plate method was conducted using mannitol salt agar for *Staphylococcus* spp., MacConkey agar for coliforms, fecal coliform agar for fecal coliforms, and selective *Streptococcus* agar for isolation group A streptococci (Difco Laboratories). We investigated *Staphylococcus* spp. and coliforms because previous studies had found them in abundance inside CAFOs (Gibbs et al. 2004; Lenhart 1982; Scarpino and Quinn 1998). After pressing of the selective media, TSA was used as a final control for the method, being pressed first and last to ensure that the organisms were being completely transferred to all plates. All plates were incubated at 35°C and counted at 24 and 48 hr. We further confirmed the presence of *Staphylococcus aureus* using Bacto coagulase plasma (Fisher Scientific, Houston, TX). We performed the replica plate method using aseptic techniques. After counting, the plates were refrigerated in an inverted position at 4°C until they were ready to be transferred onto TSA slants to be used for the Kirby-Bauer disk diffusion method.

### Antimicrobial susceptibility testing

We used the Kirby-Bauer disk diffusion method to determine the antibiotic-resistant characteristics of the recovered organisms (Bauer et al. 1966). Three Mueller-Hinton agar plates and three TSA plates were brought to room temperature and dried for each microorganism to be tested for antibiotic resistance. The TSA plates were used to ensure purity of the micro-organisms. A sterile cotton swab was used to transfer several colonies of the microorganism from the slant to a sterile saline tube until the tube was the same turbidity as the 0.5 McFarland standard under examination. This gave an estimated 10^8^ cfu/mL. The Kirby-Bauer disk diffusion method was then performed with aseptic techniques. The plates were checked for susceptibility after 24 hr. The zones of inhibition were recorded for all of the plates and then compared with the standard [National Committee for Clinical Laboratory Standards (NCCLS) 1997]. We then determined whether the microorganism was susceptible, intermediately resistant, or resistant to each antibiotic evaluated. Table 1 provides the specific NCCLS zone diameters used to categorize *S. aureus*, group A streptococci, fecal coliforms, and total coliforms as susceptible, intermediate, or resistant.

Six types of antibiotic susceptibility test disks (Difco Laboratories) were used in the Kirby-Bauer method. All six drugs (20 μg oxytetracycline, 30 μg tetracycline, 15 μg erythromycin, 10 μg ampicillin, 10 μg penicillin, and 2 μg lincomycin) are commonly used in both animal agriculture and human medicine. These six antibiotics represent four distinct classes of antibiotics. Ampicillin and penicillin are both penicillins, tetracycline and oxytetracycline are both tetracyclines, lincomycin is a lincosamide, and erythromycin is a macrolide. Multidrug resistance is defined as resistance to at least two different classes of antibiotics.

Control organisms were obtained from cultures in the environmental microbiology laboratory at the Shriner’s Burn Center (Cincinnati, OH). Control organisms (*Escherichia coli*, ATCC #25922; *Klebsiella pneumoniae*, ATCC #31488; *S. aureus*, ATCC #29213; *Streptococcus pneumoniae*, ATCC #49619; American Type Culture Collection, Manassas, VA) were used to test both the quality of the antibiotics and the media used. The control organisms were applied to the selective media to ensure that it would be able to culture the selected organism. The control organisms were also put through the Kirby-Bauer method to ensure that the antibiotics used would inhibit growth of a nonresistant culture.

### Statistical analysis

In primary analyses we used contingency table methods (3 × 4) to analyze the change in frequency of resistance, if any, associated with distance downwind from the facility. That is, the frequency distribution for the three categories of the resistance profile was compared across the four distances downwind from the facility (25, 50, 100, and 150 m). Comparisons were made regarding resistance to each antibiotic in each organism. A nonsignificant result implies that distributions of frequencies were relatively constant as distance changed. For secondary analyses, contingency table methods (2 × 2) were also used to compare frequencies at each distance downwind to the 25 m upwind frequencies (e.g., 25 m upwind vs. 25 m downwind, 25 m upwind vs. 50 m downwind). A nonsignificant result from these tests implies that frequencies of organisms at a downwind location were not different from upwind. All *p*-values were calculated using Fisher’s exact methods because many cell counts were zero and expected frequencies were < 5. Even though the Andersen two-stage samplers separate particles according to their size (non-respirable and respirable), the analyses were performed only for total organisms because of the low numbers of some selected organisms.

## Results

**Figure 1:**
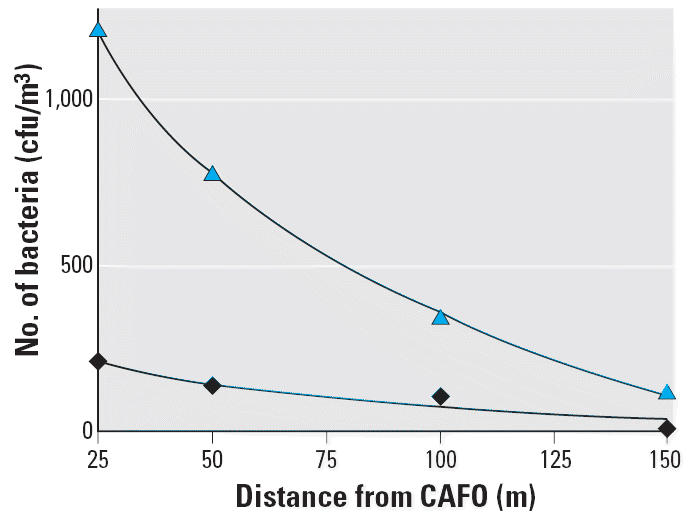
Estimated number of culturable multidrug-resistant bacteria located downwind of the facility. Triangles, cfu/m^3^ resistant to at least two classes of antibiotics [*y* = –612.25ln(*x*) + 3171.8]; diamonds, cfu/m^3^ resistant to all four classes of antibiotics [*y* = –98.936ln(*x*) + 528.33].

The summary results of the sampling are presented in Table 2. The total number of organisms found within the facility was 287 times higher than the number recovered upwind of the facility. This number decreased downwind of the facility as far as 150 m downwind (the farthest downwind sampling distance in this study), where the number of organisms was only 2.2 times higher than that recovered upwind of the facility. The percentage of organisms resistant to at least two classes of the antibiotics was 2.1 times higher inside of the facility than upwind of the facility. This percentage decreased slightly downwind of the facility; however, none of the percentages of resistance downwind of the facility was statistically different from any other (*p* > 0.05). This indicated that out to 150 m downwind, the percentage of organisms resistant to at least two classes of the antibiotics did not change. The percentage of organisms resistant to all four classes of the antibiotics evaluated was three times higher inside the facility than upwind of the facility. This immediately decreased downwind of the facility to a percentage similar to the upwind value, and none of the percentages of resistance downwind of the facility was statistically different from any other (*p* > 0.05) (Table 2). Figure 1 shows the logarithmic decrease in multidrug-resistant bacteria downwind of the CAFO.

As previously reported by Green et al. (2006), *S. aureus* was the most prevalent organism sampled, accounting for 76% (1.4 × 10^4^ cfu/m^3^; SD, 8.9 × 10^3^ cfu/m^3^) of the bacteria recovered inside of the CAFO. The percent resistant organisms increased from upwind values inside of the facilities for all antibiotics evaluated with the exception of ampicillin, which did not change. *S. aureus* was the only organism evaluated for which the decreased concentrations with increased distance downwind of the facility were statistically significant (Table 3). *S. aureus* showed this statistically significant relationship with distance from the facility and resistance profile for four of the antibiotics evaluated: oxytetracycline (*p* = 0.010), tetracycline (*p* = 0.014), ampicillin (*p* = 0.007), and erythromycin (*p* = 0.035); however, this relationship was not seen with lincomycin or penicillin (*p* > 0.05). Secondary analysis of *S. aureus* also showed a difference in resistant bacteria between upwind values and those for immediately downwind (25 m) for resistance to oxytetracycline, tetracycline, ampicillin, and erythromycin (*p* > 0.05); however, this relationship was not observed with lincomycin or penicillin (*p* > 0.05).

The percentage of resistant group A streptococci increased within the facility compared with upwind values for all antibiotics evaluated except lincomycin (Table 4). The percentage of resistant group A streptococci was not statistically different at any of the downwind distances (*p* > 0.05), and all downwind values were similar to the upwind values (*p* > 0.05) (Table 4).

The percentage of resistant fecal coliform organisms increased within the facility compared with upwind values for all antibiotics evaluated except lincomycin (Table 5). The percentage of resistant fecal coliform organisms was not statistically different for any of the downwind distances (*p* > 0.05), and all downwind values were similar to the upwind values for all antibiotics except lincomycin (*p* = 0.011) (Table 5).

The percentage of resistant total coliform organisms increased within the facility compared with upwind values only for oxytetra-cycline and tetracycline (Table 6). The percentage of resistant total coliform organisms was not statistically different for any of the downwind distances (*p* > 0.05), and all downwind values were similar to the upwind values for all antibiotics, with the exceptions of lincomycin and penicillin, which could not be evaluated statistically (Table 6).

## Discussion

This study was conducted over a month during the summer of 2003 in the American Midwest in conjunction with a previously published study (Green et al. 2006). In the present study, we consistently found bacteria that exhibited multiple antibiotic resistances to at least two classes of the study antibiotics. In a previous study (Gibbs et al. 2004), we demonstrated that the animals within the CAFO were responsible for the density of organisms released from the facility and the source of the antibiotic-resistant organisms. We also checked for patterns in multiple antibiotic resistances for all strains of bacteria isolated. In the present study, we found multiple antibiotic resistance present out to 150 m from the CAFO; these percentages were significantly higher than those recovered upwind of the facility (Table 2) and could affect employee health. It is important to note that in the previously published study the animals were currently receiving subtherapeutic antibiotics (Gibbs et al. 2004), whereas in this study the animals had received nontherapeutic doses of antibiotics 4 weeks before sampling. This would seem to indicate that antibiotic-resistant bacteria have been selected as a result of the use of the nontherapeutic levels of oxy-tetracycline and are persisting in the swine environment even after use has ceased. This is in agreement with the findings of Manson et al. (2004) and Johnsen et al. (2005).

As in previous studies (Chapin et al. 2005; Gibbs et al. 2004; Predicala et al. 2002), *Staphylococcus* was one of the most prevalent culturable genera of bacteria recovered from swine CAFOs, and it exhibited multiple antibiotic resistances. *S. aureus* in the present study had multiple antibiotic resistances throughout the distances examined (Table 3). Chapin et al. (2005) found that *Staphylococcus* spp. accounted for 32% of the organisms they recovered. This is significantly less than the 76% recovered in this study and 84.1% recovered by Predicala et al. (2002). However, this difference could be due to the different collection methods: we and Predicala et al. (2002) used impaction methods, whereas Chapin et al. (2005) used all-glass impingers. It is possible that the all-glass impingers provided better collection of other organisms or less collection of *Staphylococcus* spp. compared with the impaction collectors (Jensen et al. 1992). However, the impinger collectors did not provide size differentiation. Both Predicala et al. (2002) and Chapin et al. (2005) used media other than TSA: Predicala et al. (2002) placed R2A agar in Andersen samplers; and Chapin et al. (2005) used mE agar for the isolation of *Enterococcus* isolates and tested each isolate for the production of catalase in the presence of 3% hydrogen peroxide. Catalase-positive isolates were then identified as *Staphylococcus* species. The differences in collection media used could also account for the variability in *Staphylococcus* spp. recovery. The continued recovery of large densities of *S. aureus* from the bioaerosols indicates that future research should focus more effort on culturable and nonculturable *S. aureus*, as well as other important human pathogens. This study and the others discussed (Chapin et al. 2005; Predicala et al. 2002) examined only culturable bacterial organisms; the lack of examination of other nonculturable bacteria is a limitation of the studies. The inclusion of nonculturable bacteria may change the levels of multiple antibiotic resistances, which will have to be examined in a separate study.

In the present study, an estimated 17,000 of the 18,000 cfu/m^3^ released from the CAFO were defined as multidrug-resistant or multiple-antibiotic–resistant organisms because they were resistant to at least two classes of antibiotics. By comparison, the air located upwind of the CAFO contained an estimated 28 cfu/m^3^ that were multidrug resistant. Approximately 8,200 cfu/m^3^ recovered from inside the CAFO were resistant to all four classes of antibiotics evaluated, whereas 8.8 cfu/m^3^ recovered upwind showed the same level of resistance. This shows that individuals who work inside the facility or live in proximity downwind of the facility face a greater exposure to multidrug-resistant organisms, which could potentially affect human health.

Green et al. (2006) estimated that the bacterial concentration downwind of the facility would equal the upwind concentration at approximately 175 m from the facility. Similar predictions can be made with the multidrug-resistant bacterial concentration. Figure 1 shows the logarithmic decrease in multidrug-resistant bacteria downwind of the CAFO. This indicates that those within 175 m downwind and inside the facility receive a greater exposure to multidrug-resistant organisms than those upwind of the facility.

Both the increase in percentage and quantity of multidrug-resistant bacteria inside and downwind of the facility support Green et al.’s (2006) statement that these facilities could pose a hazard to persons in direct proximity to them. This would include those employed at the facility and those who live in close proximity to the facility. This potential health hazard exists independently of a halt in subtherapeutic treatment.

## Conclusions

Bacterial concentrations with multiple antibiotic resistances or multidrug resistances were routinely recovered inside and up to 150 m downwind of this facility at higher percentages than upwind of the facility. Subsequent numbers of multiple-antibiotic–resistant bacteria are almost three orders of magnitude higher inside the facility compared with upwind. These elevated concentrations persist to (at least) 150 m downwind of the facility. Our findings indicate that bacterial concentrations with multiple antibiotic resistances are found within and downwind of CAFOs even after subtherapeutic doses of antibiotics are removed from the animal feed. Those working at or inside the facility and those living in close proximity downwind of the facility could be at risk for adverse human health effects associated with exposure to large numbers of multidrug-resistant organisms.

## Figures and Tables

**Table 1:** NCCLS zone diameters used to categorize all organisms recovered as susceptible, intermediate, or resistant.

			Zone diameter interpretive standards (mm)^a^		
Antimicrobial agent	Disk potency	Organism	Resistant	Intermediate	Susceptible
Ampicillin	10 μg	*S. aureus*	≤ 28	[Table-fn tfn1-ehp0114-001032]—	≥ 29
		Group A streptococci	≤ 18	19–25	≥ 26
		Fecal coliforms	≤ 13	14–16	≥ 17
		Total coliforms	≤ 13	14–16	≥ 17
Erythromycin	15 μg	*S. aureus*	≤ 13	14–22	≥ 23
		Group A streptococci	≤ 15	16–20	≥ 21
		Fecal coliforms	≤ 13	14–22	≥ 23
		Total coliforms	≤ 13	14–22	≥ 23
Lincomycin	2 μg	*S. aureus*	≤ 14	15–20	≥ 21
		Group A streptococci	≤ 15	16–18	≥ 19
		Fecal coliforms	≤ 12	13–16	≥ 17
		Total coliforms	≤ 12	13–16	≥ 17
Oxytetracycline	20 μg	*S. aureus*	≤ 12	13–16	≥ 17
		Group A streptococci	≤ 14	15–18	≥ 19
		Fecal coliforms	≤ 14	15–18	≥ 19
		Total coliforms	≤ 14	15–18	≥ 19
Penicillin	10 μg	*S. aureus*	≤ 28	[Table-fn tfn1-ehp0114-001032]—	≥ 29
		Group A streptococci	≤ 19	20–27	≥ 28
		Fecal coliforms	≤ 14	[Table-fn tfn1-ehp0114-001032]—	≥ 15
		Total coliforms	≤ 14	[Table-fn tfn1-ehp0114-001032]—	≥ 15
Tetracycline	30 μg	*S. aureus*	≤ 14	15–18	≥ 19
		Group A streptococci	≤ 18	19–22	≥ 23
		Fecal coliforms	≤ 14	15–18	≥ 19
		Total coliforms	≤ 14	15–18	≥ 19

— , not detected.

a Standards adapted from NCCLS (1997, 2000, 2001).

**Table 2:** Summary of antibiotic resistance for all organisms recovered.

Organisms	25 m upwind	Inside facility	25 m downwind	50 m downwind	100 m downwind	150 m downwind
Percent resistant to all four antibiotic classes	14	45	16	14	24	10
Percent resistant to at least two classes of antibiotics	44	94	93	80	82	81
No. recovered and tested for antibiotic resistance	59	69	45	49	33	21
Average no. recovered (cfu/m^3^)	63	18,132	1,295	970	414	141

**Table 3:** *S. aureus* antibiotic resistance profile.

	25 m upwind	Inside facility	25 m downwind	50 m downwind	100 m downwind	150 m downwind
No. of organisms	11	18	14	19	20	9
Oxytetracycline						
%R	36	83	93	84	50	44
%S	55	11	7	11	45	56
%I	9	6	0	5	5	0
Tetracycline						
%R	36	89	86	84	50	56
%S	64	11	7	11	50	44
%I	0	0	7	5	0	0
Ampicillin						
%R	73	72	21	42	75	56
%S	27	28	79	58	25	44
%I						
Erythromycin						
%R	64	72	100	84	65	67
%S	27	17	0	16	35	22
%I	9	11	0	0	0	11
Lincomycin						
%R	82	94	93	95	90	78
%S	0	6	7	5	5	22
%I	18	0	0	0	5	0
Penicillin						
%R	64	83	79	63	80	89
%S	36	17	21	37	20	11
%I	0	0	0	0	0	0

Abbreviations: %I, percentage of organisms intermediately resistant; %R, percentage of organisms resistant; %S, percentage of organisms susceptible.

**Table 4:** Group A streptococci antibiotic resistance profile.

	25 m upwind	Inside facility	25 m downwind	50 m downwind	100 m downwind	150 m downwind
No. of organisms	2	19	12	14	9	4
Oxytetracycline						
%R	50	100	67	64	67	75
%S	50	0	25	22	33	25
%I	0	0	8	14	0	0
Tetracycline						
%R	50	100	67	57	67	100
%S	50	0	33	7	22	0
%I	0	0	0	36	11	0
Ampicillin						
%R	50	74	17	43	45	50
%S	50	26	66	57	44	50
%I	0	0	17	0	11	0
Erythromycin						
%R	50	68	67	57	67	75
%S	50	21	33	29	22	25
%I	0	11	0	14	11	0
Lincomycin						
%R	100	100	92	79	89	75
%S	0	0	8	14	0	25
%I	0	0	0	7	11	0
Penicillin						
%R	50	74	50	29	44	50
%S	50	10	33	50	56	50
%I	0	16	17	21	0	0

Abbreviations: %I, percentage of organisms intermediately resistant; %R, percentage of organisms resistant; %S, percentage of organisms susceptible.

**Table 5:** Fecal coliform antibiotic resistance profile.

	25 m upwind	Inside facility	25 m downwind	50 m downwind	100 m downwind	150 m downwind
No. of organisms	13	17	13	11	3	6
Oxytetracycline						
%R	38	94	62	36	67	33
%S	54	6	38	64	33	67
%I	8	0	0	0	0	0
Tetracycline						
%R	38	88	54	36	67	50
%S	54	6	38	55	33	50
%I	8	6	8	9	0	0
Ampicillin						
%R	85	65	62	36	33	83
%S	15	35	30	64	67	17
%I	0	0	8	0	0	0
Erythromycin						
%R	69	64	84	46	33	67
%S	23	18	8	36	33	33
%I	8	18	8	18	34	0
Lincomycin						
%R	100	94	100	100	67	66
%S	0	6	0	0	33	17
%I	0	0	0	0	0	17
Penicillin						
%R	69	65	92	73	33	83
%S	31	35	8	27	67	17
%I	0	0	0	0	0	0

Abbreviations: %I, percentage of organisms intermediately resistant; %R, percentage of organisms resistant; %S, percentage of organisms susceptible.

**Table 6:** Total coliform antibiotic resistance profile.

	25 m upwind	Inside facility	25 m downwind	50 m downwind	100 m downwind	150 m downwind
No. of organisms	10	16	6	7	—	2
Oxytetracycline						
%R	10	75	0	14	—	50
%S	90	19	100	86	—	50
%I	0	6	0	0	—	0
Tetracycline						
%R	10	69	0	0	—	50
%S	90	18	100	71	—	50
%I	0	13	0	29	—	0
Ampicillin						
%R	80	81	50	71	—	100
%S	20	19	33	29	—	0
%I	0	0	17	0	—	0
Erythromycin						
%R	70	63	33	71	—	0
%S	30	37	17	0	—	100
%I	0	0	50	29	—	0
Lincomycin						
%R	100	100	100	100	—	100
%S	0	0	0	0	—	0
%I	0	0	0	0	—	0
Penicillin						
%R	90	88	100	100	—	100
%S	10	12	0	0	—	0
%I	0	0	0	0	—	0

Abbreviations: —, not detected; %I, percentage of organisms intermediately resistant; %R, percentage of organisms resistant; %S, percentage of organisms susceptible.
